# Supplementary material for: A blended wavefield separation method for seismic exploration based on improved GoogLeNet
Source: PLoS One. 2024 Jun 25;19(6):e0304207. doi: 10.1371/journal.pone.0304207 (PMC11198824; doi:10.1371/journal.pone.0304207)
Supplement: Attachment — Submitted filename: response to reviewers.pdf [file pone.0304207.s001.pdf]

Dear Editors/ Reviewers:

We appreciate the constructive feedback provided by the reviewers and editors, and have carefully addressed each comment in this revised version. In response to the reviewers' comments, we have made several significant improvements to the manuscript, including:

Reviewer #1:

1. In this paper, the authors proposed "A blended wavefield separation method for seismic exploration based on deep learning". However, a lot of research has been studied on the application of deep learning methods in the blended seismic data. The title does not reflect the innovative points, but only repeats the results of the previous work. Therefore, this paper does not meet the requirement of the published paper.

Response:We have modified the title of the article to "A blended wavefield separation method for seismic exploration based on improved GoogLeNet" to provide more appropriateness with the content of the article.

2. In addition, it is suggested that the authors should study the characteristics of blended seismic data, and design targeted neural networks to solve the problem of the blended seismic data.

Response:We thoroughly reorganized and extensively analyzed the characteristics and mathematical representation of blended seismic data, and described the proposed deblending model based on improved GoogLeNet in detail in "Blended wavefield separation method" section of the paper to addressing the concerns raised by Reviewer #1.

Reviewer #2:

1. The source and acquisition procedures of the data used in the study should be included in detail. Was this data taken from an open data set? If it was collected by the study team, where and when was it obtained? Has it been subjected to any pre-exclusion? From how many different sources was it obtained? The article is expected to answer these and similar questions.

Response: The dataset used in this paper were downloaded from SEG WiKi and are available for public use, which provided the data by Petroleum Geo-Systems (PGS) for the 2017 IEEE signal processing competition in Tokyo (2017). After downloading the dataset, we reorganized the dataset.The reorganized dataset includes a blended data matrix and an unblended data matrix.The size of the each data matrix is 2768×256×1×256. The first dimension (of size 2768) corresponds to time samples. The second and fourth dimensions (of size 256) correspond to receiver and source indexes, and the third dimension (of size 1) is reserved. In the dataset, the time intervals between 2 or more sources in a shot-gather record are sometimes reduced to an extent that the time between two sources is less than the time it takes for all reflections caused by a source to dampen.

The above information has been elaborated in the "data vaditation –dataset processing" part of the article.

2. Considering the limited number of data used in the study, have measures been taken against the problem of overfitting? Is there a possibility that the deep learning network may have memorized?

Response： Aiming at the limited number of data used in the study, the seismic data is divided into 48×96 sized data blocks before training. On the one hand, the hardware requirements in the training process can be reduced, and on the other hand, the number of training data samples can be increased. At the same time, combined with the characteristics of training samples, the Inception model in GoogLeNet is improved to simplify the model structure and avoid overfitting.

3. Within the scope of the study, the data set is divided into three as training, validation and testing. Since the dataset used in the study is small, cross-validation is recommended.

Response: As mentioned before, the the seismic data is divided into 48×96 sized data blocks before training, and the number of training samples is 71688, the number of validation or testing is 8700.According to Reviewer #2's suggestion, we compared the training accuracy,validation accuracy and the accuracy of the test data .These values are basically the same. Therefore, the method of cross validation is not used in this paper.

4. If data obtained from a single source is used, it is recommended to verify with a different source in order to generalize the results. For example, if two seismic sources are used in the dataset, what will be the result when a blended signal with four sources is encountered?

Response:The dataset used in this paper were downloaded from SEG WiKi and are available for public use, which provided the data by Petroleum Geo-Systems (PGS) for the 2017 IEEE signal processing competition in Tokyo (2017). After downloading the dataset, we reorganized the dataset.The reorganized dataset includes a blended data matrix and an unblended data matrix.The size of the each data matrix is 2768×256×1×256. The first dimension (of size 2768) corresponds to time samples. The second and fourth dimensions (of size 256) correspond to receiver and source indexes, and the third dimension (of size 1) is reserved. In the dataset, the time intervals between 2 or more sources in a shot-gather record are sometimes reduced to an extent that the time between two sources is less than the time it takes for all reflections caused by a source to dampen.

The above information has been elaborated in the "data vaditation –dataset processing" part of the article.

Reviewer #3:

1.Deblending is a very specific task in seismic exploration workflow. So, maybe it would be better to dig into the task itself rather than a very general perspective about seismic data importance. A big portion of the manuscript is dedicated to the basics of the seismic surveys.

Response: The introduction section of the paper has been modified to elucidate the industrial demand for blended seismic wavefield separation and the current research status of data deblending by deep learning method, aiming to demonstrate the necessity and significance of our study.

2- Again the authors in paragraph 2 of the introduction talk about the basics of the ML. The manuscript should be sharp and to the point. Any literature review should be relevant to the context and its detail should be useful.

Response : The introduction section of the paper has been modified to elucidate the industrial demand for blended seismic wavefield separation and the current research status of data deblending by deep learning method, aiming to demonstrate the necessity and significance of our study.

3- The authors start with chronological literature review of the ML for deblending. Why chronological? Do you think each author followed the path of the previous ones. It doesn't seem that way. The way the papers are cited is completely wrong. Also, at some point (line 46 onward) the authors decided not to continue chronologically, why?

Response :The introduction section of the paper has been modified to elucidate the industrial demand for blended seismic wavefield separation and the current research status of data deblending by deep learning method, aiming to demonstrate the necessity and significance of our study.

4- The authors keep using misleading phrases. It is constantly mentioned in the manuscript that the GoogLeNet was improved. Do you mean you calibrated/tailored/fine-tuned the GoogleNet for your purpose? A process that is usually referred to as Transfer Learning in ML world is not improvement.

Response:We thoroughly reorganized and extensively analyzed the characteristics and mathematical representation of blended seismic data, and described the proposed deblending model based on improved GoogLeNet in detail in "Blended wavefield separation method" section of the paper.

5- This is the first manuscript I have seen that includes two INTRODUCTION chapters.

Response: delete some contents of the second section which named "Basic principle" to simply the paper.

6- The basic principles section of the second introduction includes very basic principles of blended acquisition. Do you think this is interesting for an audience. There are reference books to learn about blended acquisition.

Response: delete some contents of the second section which named "Basic principle" to simply the paper.

7- Where is the data chapter? Where does the data come from? Very bad quality pictures with no descriptions. For example, Figure 5, where are the axis. What are the plots?

Response: The dataset used in this paper were downloaded from SEG WiKi and are available for public use, which provided the data by Petroleum Geo-Systems (PGS) for the 2017 IEEE signal processing competition in Tokyo (2017). We reorganized and uploaded the dataset.The reorganized dataset includes a blended data matrix and an unblended data matrix.The size of the each data matrix is 2768×256×1×256. The first dimension (of size 2768) corresponds to time samples. The second and fourth dimensions (of size 256) correspond to receiver and source indexes, and the third dimension (of size 1) is reserved. In the dataset, the time intervals between 2 or more sources in a shot-gather record are sometimes reduced to an extent that the time between two sources is less than the time it takes for all reflections caused by a source to dampen.

The above information has been elaborated in the "data vaditation –dataset processing" part of the article.

8- From what I see from the left panel in Figure 5, the data are not even blended. It seems the shootings were not even close in time to cause blending of the wavefields.

Response: In order to verify the blended wavefield separation effect of the proposed model based on improved GoogLeNet, the network models commonly used in deep learning such as AlexNet, VGG-16, VGG-19, UNET and original GoogLeNet are selected for comparative experiments.The parameters of different models, the vadidation RMSEcurves in the training process and the quantitative evaluation index of the de-blending results in the test dataset are also supplemented . Through the detailed comparative analysis of different models in the training process and the performance in the test dataset, the performance and effectiveness of the proposed blended wavefield separation model based on improved GoogLeNet are further validated.

9- What is the value in equation 11? Do you mean you normalize the data to gray scale? Your equation does not reflect that.

Response: In the data conversion calculation process, the "value" in the equation is 1.

10- What is the benchmark method? How the deblended data is labeled?

Response: The dataset used in this paper were downloaded from SEG WiKi and are available for public use, which provided the data by Petroleum Geo-Systems (PGS) for the 2017 IEEE signal

processing competition in Tokyo (2017). We reorganized and uploaded the dataset.The reorganized dataset includes a blended data matrix and an unblended data matrix.

11- The authors say the misfit doesn't change after 40 epochs. It seems they reach a local minimum much earlier.

Response: In this paper, the experimental content is added, and the analysis method of training results is also optimized.Please see "Data vadidation-Test results"section of the revised paper.

12- What is figure 8, 9. No description. No interpretation.

Response: In this paper, the experimental content is added, and the analysis method of training results is also optimized.Please see "Data vadidation-Test results"section of the revised paper.

13- In conclusions the authors say: "Consequently, traditional filtering algorithms employed in seismic data processing fail to yield satisfactory outcomes, particularly in scenarios where ambient noise is prevalent." Where is this information in the paper? I don't see any comparison. Benchmark method or traditional method.

Response: We have modified and expanded the conclusion sectionto provide a more comprehensive interpretation of our results and their implications for the field.

is modified to be more appropriate for the design and implementation of the aliasing separation model based on the improved googlenet network

14- Again, in conclusion, the authors compare the performance of their application with other algorithms that were not shown.

Response :In this revised paper, the comparative deblending results with AlexNet, VGG-16,VGG-19,U-Net，GoogLeNet are added.

15- In addition to these comments that are summarized the language of the paper is significantly poor. Some of the phrases are misleading and some can not be understood.

Response: we recheck syntax and description, hope to make the paper more clear and easy to understand.

Thanks for your time and consideration of our manuscript. We look forward to hearing from you regarding the outcome of the review process.

Sincerely,

ZhiQiang Gan，Xiang-E Sun
Yangtze Univerisy
