# Supplementary material for: A blended wavefield separation method for seismic exploration based on improved GoogLeNet
Source: PLoS One. 2024 Jun 25;19(6):e0304207. doi: 10.1371/journal.pone.0304207 (PMC11198824; doi:10.1371/journal.pone.0304207)
Supplement: Attachment — Submitted filename: response to reviewers2.pdf [file pone.0304207.s002.pdf]

Dear Alberto Marchisio and Reviewers:

Thank you very much for your careful review of my manuscript and for providing valuable feedback. Your comments have been extremely helpful in improving the quality of the paper. Below, I have addressed each of the reviewers' comments in detail:

Reviewer #3 Comments

1- There is still no proper data section. The readers cannot be expected to go to the SEG wiki, find the data manual from PGS, and understand how the data were acquired and what the details are.

Response: Upon careful consideration, We added a [Data availability and access] section to the article. In this section, we describe the source of the data and related details. The datasets used in our paper can be downloaded from the URL: https://doi.org/10.17605/OSF.IO/VW6A3.

2- In Figures 5 and 6, what is the value (V)? Do you mean amplitude?

Response:This "value (V)" represents the true value of the data, and the ' V ' in parentheses is the unit of the true value, volt.

3- In your response, you mentioned that the value in equation 8 corresponds to one. You did not provide further clarification. If it is one, then why is it there?

Response: In this paper, seismic data is processed as a special grayscale image, and seismic data is a data with a large dynamic range, and the "value " in equation 8 needs to be determined according to the maximum and minimum values of the data to be processed. The range of it is also mentioned in equation 8 , which is 1-255, and the value 1 is selected this time. In other application scenarios, other values in the range [1,255] may be selected according to the data characteristics.

4- The authors keep mentioning theoretically deblended data. What do you mean by theoretical blended data? Do you mean the benchmark deblended data by PGS? You have to mention how it was obtained.

Response:metion in section*[Data availability and access]*. The theoretically deblended data used in this paper were obtained using a deblending method that removes simultaneous source interference by utilizing shot delays in the time-delay matrix to locate and remove unwanted shots from the data matrix, which is also provided in the PGS package.

5- Stressing on the data, one after reading the manuscript will not understand if the data are synthetic or real data. This important information should be embedded in the manuscript.

Response: The blended dataset supporting the findings of this study was the real data of seismic survey provided by PGS and is available for public use.

Reviewer #4 Comments

The novelty is incremental. Most of the spce is given to the well known facts and thery. ai is mentioned that imporved goolgenet is used but the improvement is not obvious. Its just using neural network to carry the already well studied deblending task.

Response: This article innovatively constructs a mathematical model for the separation of blended wavefields based on deep learning theory, addressesing the ill-posed equation solving problem in the separation of blended wavefields. Furthermore, according to the characteristics of seismic data, the effectiveness of the proposed model

is validated based on an improved GoogLeNet network. Compared to several other commonly used deep learning networks, the enhanced GoogLeNet not only achieves better deblending effects but also significantly reduces the hardware requirements. The authors consider this approach a significant advancement in the application of AI methods in the field of seismic exploration.

Reviewer #5 Comments

I did not review the manuscript in the first round; instead, my assessment is based on analyzing the responses to other reviewers' comments from the initial round. I noticed that the authors have made substantial changes compared to the original version, and their responses in the rebuttal letter are generally sound. However, I noticed a minor issue where a few responses appeared to be verbatim repetitions and, in some cases, not directly addressing the specific concerns raised by the reviewers. Aside from this observation, I have no further comments and recommend the manuscript for publication.

Response: Thank you for your thorough evaluation of the manuscript, and we have further addressed the reviewers' concerns.

Thanks for your time and consideration of our manuscript. We look forward to hearing from you regarding the outcome of the review process.

Sincerely,

ZhiQiang Gan，Xiang-E Sun

Yangtze University
